# Multifunctional Thioredoxin-Like Protein from the Gastrointestinal Parasitic Nematodes* Strongyloides ratti* and* Trichuris suis* Affects Mucosal Homeostasis

**DOI:** 10.1155/2016/8421597

**Published:** 2016-10-31

**Authors:** Dana Ditgen, Emmanuela M. Anandarajah, Jan Hansmann, Dominic Winter, Guido Schramm, Klaus D. Erttmann, Eva Liebau, Norbert W. Brattig

**Affiliations:** ^1^Department of Molecular Physiology, Westfälische Wilhelms-University, Münster, Germany; ^2^Department of Molecular Medicine, Bernhard Nocht Institute for Tropical Medicine, Hamburg, Germany; ^3^Department of Tissue Engineering and Regenerative Medicine (TERM), University of Würzburg, Germany; ^4^Institute for Biochemistry and Molecular Biology, University of Bonn, Bonn, Germany; ^5^Ovamed GmbH, Hamburg, Germany

## Abstract

The cellular redox state is important for the regulation of multiple functions and is essential for the maintenance of cellular homeostasis and antioxidant defense. In the excretory/secretory (E/S) products of* Strongyloides ratti* and* Trichuris suis* sequences for thioredoxin (Trx) and Trx-like protein (Trx-lp) were identified. To characterize the antioxidant Trx-lp and its interaction with the parasite's mucosal habitat,* S. ratti* and* T. suis* Trx-lps were cloned and recombinantly expressed. The primary antioxidative activity was assured by reduction of insulin and IgM. Further analysis applying an* in vitro* mucosal 3D-cell culture model revealed that the secreted Trx-lps were able to bind to monocytic and intestinal epithelial cells and induce the time-dependent release of cytokines such as TNF-*α*, IL-22, and TSLP. In addition, the redox proteins also possessed chemotactic activity for monocytic THP-1 cells and fostered epithelial wound healing activity. These results confirm that the parasite-secreted Trx-lps are multifunctional proteins that can affect the host intestinal mucosa.

## 1. Introduction

Parasitic intestinal nematodes are widespread, affecting human and vertebrates. Worldwide, more than one-third of mankind is infected with helminths [1] of which 100–200 million people are infected with* Strongyloides *[2, 3] and approximately 800 million with* Trichuris* [4]. The investigated nematodes* Strongyloides ratti *and* Trichuris suis* are very closely related to their human-pathogenic homologues* Strongyloides stercoralis *and* Trichuris trichiura* [5, 6].

In contrast to immune responses to microbes with mainly inflammation, the immune responses to helminths are mostly less intense and highly regulated [7]. Modulation of the host's immune response reported from* T. suis* ova can be beneficial for an attenuation of inflammatory bowel diseases (IBD) such as Crohn's disease and ulcerative colitis [8, 9]. Helminths release multiple excretory/secretory (E/S) products which enable them to establish, survive, and reproduce in their hosts successfully [10, 11]. In case of* S. ratti* and* T. suis*, these E/S products include antioxidative proteins such as thioredoxin (Trx), heat shock proteins, and numerous proteases as well as protease inhibitors, galectins, and orthologous of host cytokines [10, 12–16]. Trx has also been reported in E/S products of multiple helminths [17–20]. Recently, these E/S proteins have also been detected in extracellular vesicles from helminths [21].

Trx or the Trx system in general is widespread from archaea to human consisting of Trx, the Trx reductase, and NADPH [22]. Hereby, Trx is reduced by the Trx reductase in an NADPH-dependent manner [23]. In general, Trx superfamily members regulate thiol-based redox control, operating as protein disulfide oxidoreductases, and protect cytosolic proteins against aggregation in the cell [24]. Its redox-regulating activity is important for DNA replication, maintenance of the cellular redox state, and, therefore, the cellular homeostasis and antioxidant defense [22, 25]. Furthermore, Trx is part of multiple cellular pathways [26] and capable of regulating transcription factor activities, inhibition of apoptosis, protection from high-energy oxygen radicals, and regeneration of denatured proteins and is critical for signal transduction through thiol redox control as well as more specific processes like presenting antigens [22, 23, 26–28]. Without a signal peptide, Trx is secreted by a nonclassical secretory pathway by various cells [29, 30].

The numerous extracellular activities of Trx include anti-inflammatory and antiapoptotic, and thus cytoprotective effects [31–33]. Of interest, multifunctional prokaryotic Trx, which displays unrelated properties, that is, antioxidant activity and promotion of DNA replication, has been described as moonlighting protein [34–36]. In the E/S products of* Strongyloides* and of multiple other helminths numerous multifunctional proteins have been detected like the moonlighting enzymes enolase and glyceraldehyde-3-phosphate dehydrogenase [10, 13, 37–39].

While Trx is well characterized, less is known about the functions of Trx-lp [26]. The Trx-lp, a member of the Trx superfamily, is a fusion protein composed of the classical Trx domain (WCGPC) at the N-terminus and a C-terminal proteasome-interacting thioredoxin (PITH) domain, formerly known as DUF1000 (protein families database, http://pfam.xfam.org/family/PF06201). It is larger than the classical Trx (12 kDa), which is highly conserved in all species [23, 25]. Proteins of the Trx superfamily have been reported in various protozoan parasites including* Plasmodium, Trypanosoma, *and* Toxoplasma* [40–43] and in the trematode* Clonorchis sinensis *[44]. Besides thiol-based redox control, eukaryotic Trx-lps are also involved in signaling processes as cofactors of certain enzymes, regulating specific signal proteins [45, 46]. For example, the human Trx-related protein (TRP32), known as TXNL-1, protects the cell against glucose deprivation-induced cytotoxicity and is involved in activation of antiapoptotic Akt/PI3K signaling as well as PTEN (phosphatase and tensin homologue deleted on chromosome ten) inhibition [47, 48]. Another example is the thioredoxin domain containing 17 (TXNDC17), also known as Trx-related protein of 14 kDa (TRP14), which is STAT-3-dependent and responsible for the drug resistance in human colorectal cancer cells. TRP14 also shows, like Trx1, S-nitrosylase activity and furthermore is able to control the TNF-*α*/NF-*κ*B signaling pathway [49–51]. In addition, PTEN is also an interaction partner of human Trx and among others Trx controls the TNF-*α*/NF-*κ*B signaling pathway as well [52, 53]. The novel thioredoxin-related transmembrane protein TMX4 is a type I transmembrane protein with its Trx-like domain inside the ER which possibly plays a role in the correct folding of proteins inside the ER due to its reductase function [54].

Since Trx have been reported to act as chemoattractant for leukocytes and to induce cytokines [31] we wanted to examine if SrTrx-lp has similar impact on monocytic cells.

In the present study we cloned and characterized two Trx-lps and investigated some functional activities including their chemotactic activity, their ability to promote wound healing processes in the intestinal epithelial cell (IEC) Caco-2 model, and their involvement in cytokine release in a three-dimensional- (3D-) cell culture model.

## 2. Material and Methods

### 2.1. Parasites

The* S. ratti* life cycle was maintained in our laboratory as reported [13, 15]. Animal experiments were approved by and conducted in accordance with guidelines of the Animal Protection Board of the City of Hamburg (G21131/591-00.33). The life cycle was maintained using Wistar rats by serial passage and the developmental stages isolated as described [14].* T. suis* stages were obtained from Ovamed (Hamburg, Germany).

### 2.2. Preparation of Somatic Extracts

*S. ratti* and* T. suis* extracts were prepared from freshly harvested life stages as described before [13, 15].

### 2.3. DNA Sequencing and Bioinformatic Analysis

PCR products and plasmids were sequenced by the dideoxy termination method of Sanger performed by eurofinsgenomics.eu. For homology searches the NCBI Blast Program was used (http://www.ncbi.nlm.nih.gov/). Further, for bioinformatics analyses the Expert Protein Analyses System (ExPASy) proteomics server of the Swiss Institute of Bioinformatics (http://expasy.org/tools/) was used. To obtain the conserved domains of the Trx-lps the protein families database (Pfam) of the USA server (http://pfam.xfam.org/family/PF06201) was used which represents proteins by multiple sequence alignments and hidden Markov models (HMMs). Multiple sequence alignments were performed by the program CLUSTAL_W2 (http://www.ebi.ac.uk/Tools/msa/clustalw2/) from the European Bioinformatics Institute which is part of the European Molecular Biology Laboratory (EMBL-EBI).

### 2.4. Mass Spectrometry

SrTrx-lp and TsTrx-lp SDS-PAGE bands were excised, cut into small cubes, and transferred to microtubes and in gel digestion was performed as described elsewhere [57]. Briefly, gel pieces were destained using 30% acetonitrile (ACN), 0.07 M NH_4_HCO_3_, reduced with 20 mM dithiothreitol and alkylated by 1% acrylamide, and dehydrated using 100% ACN [57]. ACN was removed and the gel pieces were dried using a vacuum centrifuge and rehydrated in 0.1 M NH_4_HCO_3_ containing 0.5 *μ*g of trypsin (Promega, Mannheim, Germany). A sufficient volume of 0.1 M NH_4_HCO_3_ was added to cover the gel pieces completely and digestion was performed at 37°C overnight. The peptide containing supernatant was transferred to new microtubes and the gel pieces were extracted with 50% ACN, 0.1% trifluoroacetic acid followed by 0.1 M NH_4_HCO_3_ and ACN. Samples were dried in the vacuum centrifuge, resuspended in 5% ACN and 5% formic acid, desalted using C_18_ StageTips [58], dried again, and resuspended in 5% ACN and 5% formic acid. For reversed phase chromatography in house manufactured analytical columns were used. Using 100 *μ*m inner diameter fused silica capillaries, spray tips were generated with a P2000 laser puller (Sutter Instruments, Novato, CA, USA) and packed with 5 *μ*m ReproSil-Pur 120 C_18_-AQ particles (Dr. Maisch, Ammerbuch-Entringen, Germany). Peptides were loaded directly on the analytical column using a nanoflow UHPLC system (EASY-nLC 1000, Thermo Fisher Scientific, Bremen, Germany) at a flow rate of 1 *μ*L/min solvent C (water with 0.1% formic acid). Peptides were eluted applying a 60 min linear gradient from 100% solvent A (water with 5% DMSO [59], 0.1% formic acid), to 65% solvent A, 35% solvent B (ACN with 5% DMSO, 0.1% formic acid) at a flow rate of 400 nL/min. Eluting peptides were ionized in the positive ion mode at 1.6 kV in the nanospray ion source of an Orbitrap Velos mass spectrometer (Thermo Fisher Scientific, Bremen, Germany). Survey scans (*m/z* 400 to 1200) were performed in the Orbitrap analyzer at a resolution of 30,000 followed by fragmentation of the 10 most abundant ions in the linear ion trap by collision induced dissociation. Dynamic exclusion was set to 30 sec with an exclusion list size of 500. Thermo ^*∗*^.raw files were analyzed using Maxquant (version 1.5.2.8) using the following settings: protein N-terminal acetylation and oxidation of methionine were set as variable modifications and propionamide at cysteine was set as fixed modification; enzyme specificity was set to trypsin and up to two missed cleavage sites were allowed. Data were searched against a database consisting of all* S. ratti* and* T. suis* entries from Uniprot/TrEMBL (version from 12/01/2014, 12,462 entries) as well as common contaminations. The false discovery rate was set to 1%.

### 2.5. Cloning, Expression, and Purification of Recombinant Trx-lps

*S. ratti* and* T. suis* RNA were isolated from adult parasitic females as described before [15] and the cDNA was synthesized by using the First Strand cDNA Kit from New England BioLabs® Inc. according to the manufacturer's instructions. Forward and reverse primers were generated using the online tool provided by Clontech (http://bioinfo.clontech.com/infusion/) (TsTrx-lp: forward: AAGGTCGTCATATGATGGCT ATAAAGGAGATAA; reverse: TCCTCGAGAATTCCTAATGAGCTTCTCCCTT; SrTrx-lp: forward: AAGGTCGTCATATGATGGCTATAAAGGAGATAA; reverse: TCCTCGAGAATTCCTAATGAGCTTCTCCCTT). Fragments were amplified by PCR using the InFusion® HD Cloning Kit from Clontech according to the manufacturer's instructions and the Phusion High-Fidelity DNA-Polymerase from Thermo Scientific (Waltham, USA). The Trx-lp PCR fragments from* S. ratti* and* T. suis* were cloned into pJC45 vector [60] and IBA 3 plus vector, transformed into* Escherichia coli* Stellar cells (Clontech, USA) and sequenced (eurofins MWG).

The* S. ratti* and* T. suis* Trx-lps were expressed in lipopolysaccharide- (LPS-) free* E. coli* strain ClearColi® BL21 (DE3) (Lucigen Simplifying Genomics), which do not trigger the endotoxic response in human cells, in Luria-Bertani medium containing 100 *μ*g/mL ampicillin. The expression of the His-tag fusion proteins was induced by isopropyl-*β*-D-thiogalactopyranoside (IPTG, final concentration 1 mM) and the expression of the Strep-tag fusion proteins by anhydrotetracycline (AHT, final concentration 200 *μ*g/L), for 5 h at 37°C. The bacterial cells were collected by centrifugation (6,000 ×g) for 15 min and kept at −20°C until use. Recombinant proteins were purified by using Ni^2+^ affinity chromatography (Qiagen, Hilden, Germany) or Strep-Tactin® Superflow Plus (Qiagen, Germany) according to the manufacturer's instructions. The imidazole or desthiobiotin was removed by dialysis overnight using phosphate-buffered saline (PBS, pH 7.4). Even though the endotoxin-free* E. coli* strain was used the LPS inhibitor polymyxin B (30 *μ*g/mL) was added to all buffers used. Sodium dodecyl sulfate polyacrylamide gel electrophoresis (SDS-PAGE) was applied to verify expression and purity of the proteins, which were visualized by Coomassie brilliant blue G-250 staining. The protein concentration was quantified by Bradford assay. Furthermore, the elutions were analyzed by semidry Western blot. After SDS-PAGE and the following transfer onto nitrocellulose membranes, the membranes were incubated with the anti-his6-peroxidase (2) (mouse monoclonal; 1 : 5000; Roche life science, Mannheim, Germany) overnight at 4°C.

### 2.6. Functional Activity Assays

#### 2.6.1. Insulin Reduction

According to the method of Holmgren [61] (1979) as well as Luthman and Holmgren [62] (1982), disulfide reduction activity was measured by reduction of insulin [61, 62]. In this test, the turbidity of the sample was measured, which is caused by the precipitating reduced insulin. The resulting decrease in absorbance was measured at 650 nm. During the reaction, the SrTrx-lp was repeatedly regenerated by DTT. Here, the regeneration of active Trx-lp is faster than the direct reduction of insulin by DTT. Initially, 1.6 mM insulin (bovine pancreas, Sigma-Aldrich, Hamburg, Germany) was prepared by a suspension of 50 mg of insulin in 2.5 mL 100 mM potassium phosphate buffer (pH 6.5) for the reaction approach. Here, the pH was first adjusted to 3 with 1 M HCl solution to completely dissolve the protein and the pH was adjusted to 6.5 with 1 M NaOH. The solution was supplemented with dH_2_O to a volume of 5 mL. Thereafter, a master mix of 825 *μ*L 1.6 mM insulin (160 *μ*M final volume) and 4675 *μ*L PE (100 mM potassium phosphate, 2 mM EDTA, pH 6.5) buffer was prepared. SrTrx-lp was tested at a concentration of 1 *μ*M (30 *μ*g/mL), 2.5 *μ*M (75 *μ*g/mL), and 5 *μ*M (150 *μ*g/mL). In an interval of 1 min over a period of 40 min, the reduction of insulin by SrTrx-lp was measured. As a negative control, the same reaction approach was used without redox regulatory protein. The amount of SrTrx-lp was replaced by PE-buffer. The relative specific enzymatic activity was calculated by the following formula: Δ*A*_650_ × 1000/mg protein concentration in the reaction mix.

#### 2.6.2. IgM Reduction

According to the method of Wollman et al. (1988), the Trx-lp from either* S. ratti* or* T. suis* was reduced by 100 mM DTT for 1 h at room temperature (RT) and dialyzed against 80 mM HEPES and 10 mM EDTA buffer for 1 h at 4°C to remove DTT [63]. The dialysis buffer was also used as reaction buffer. The buffer was mixed with 1.7 *μ*M IgM (PierceTM Mouse IgM Isotype Control, Thermo Scientific, Czech Republic) and 0.5 *μ*L, 1 *μ*L, and 5 *μ*L of the reduced Trx-lp solution for overnight reaction at RT. For protein size determination SDS-PAGE analysis was performed under nonreducing conditions (5–12% acrylamide gradient). Silver nitrate staining was used to visualize proteins [63].

### 2.7. Cells

#### 2.7.1. Preparation of Peripheral Blood Cells

In agreement with institutional guidelines healthy volunteers served as source for peripheral blood mononuclear cells (MNC) and polymorphonuclear cells (PMN) purified from venous blood samples (collected in sodium citrate tubes). First, erythrocytes were sedimented from anticoagulated blood samples by addition of equal amounts of 6% hydroxyethyl starch (HEAS-steril®, Fresenius, Friedberg, Germany). MNCs were separated from PMN as reported before by density centrifugation using a two-level density gradient consisting of Mono-Poly Resolving Media (1.114 g/ML; MP Biomedicals, Stockholm, Sweden) and Lymphoflot (1.077 g/mL; Bio-Rad, Dreieich, Germany) [14]. Both the MNC interphase and the PMN interphase were collected and the rest discarded. The cells were washed carefully with PBS, followed by a centrifugation step at 1,800 rpm for 10 min. This step was optionally repeated one more time, if too many platelets were present. While the MNCs were added to the THP-1 media, the PMNs were resuspended in HBSS both at a concentration of 5 × 10^5^ cells/mL and stored on ice until further use.

#### 2.7.2. Three-Dimensional Coculture

To analyze the immunological effect of SrTrx-lp and TsTrx-lp, the recombinant proteins were used as stimuli in a 3D-coculture model, composed of human intestinal epithelial and dendritic cells (DCs), derived from monocytic THP-1 cells, grown on a collagen scaffold that mimics the* in vivo* natural microenvironment [64].

The human intestinal epithelial cells, Caco-2 cells, were grown in DMEM media (with 10 % FCS, 1% nonessential amino acids, 1% Pen/Strep; Liefer-Co) until denseness of 70–80% was reached and seeded on 12-well plates in ThinCerts™ TC inserts (Greiner BioOne) followed by the addition of 200 *μ*L collagen (University Hospital Würzburg) to each insert. Prior to adding the Caco-2 cells, the collagen was incubated 1 h at 37°C for gelation. To detach the Caco-2 cells from the flask the cells were trypsinized prior to transfer 10^5^ cells/well into the collagen-layered inserts and incubated for 2 h at 37°C and 5% CO_2_ to let them adhere on the collagen. Afterwards, wells were floated with DMEM media. The cells were grown for at least 14 days until a monolayer was formed. For differentiation to DCs, THP-1 cells were washed twice in PBS and seeded in serum-free RPMI 1640 media supplemented with IL-4 (1000 IU/mL; Peprotech, Hamburg, Germany) and GM-CSF (1000 IU/mL; Peprotech) and were grown for 7–10 days [65]. Subsequently the generation of mature DCs was verified by staining 10^5^ washed cultured cells with phycoerythrin- (PE-) conjugated monoclonal anti-CD86 (B7-2) antibodies (mouse anti-human CD86-PE-conjugated antibody; Becton-Dickinson Bioscience, San Diego, USA, and a PE-conjugated isotype control; PharMingen, Leiden, Netherlands) analyzed by flow cytometry (CellQuestPro; BD) (data not shown) [66]. After proper development of both cell types, the Caco-2-collagen inserts were transferred to the wells with grown DCs, which were floated with DMEM media (10% FCS, 1% nonessential amino acids, 1% Pen/Strep).

The Trx-lps were added as stimuli (5 *μ*g, 10 *μ*g, and 25 *μ*g/mL), while the UFM-1 activating protein UBA-5 (25 *μ*g/mL) from the nonparasitic nematode* Caenorhabditis elegans* served as negative control. UBA-5 was cloned and expressed as published by our group [67]. Further controls were performed with the bacterial cell wall components LPS (1 *μ*g/mL; Sigma-Aldrich, Taufkirchen, Germany) and lipoteichoic acid (LTA, 0.1 *μ*g/mL; Sigma-Aldrich, Taufkirchen) to analyze potential endotoxin contaminations and to compare both responses. Worm extract from* T. suis* served as positive controls for a T_H_2 response. The supernatants were taken after 24 h, 48 h, and 72 h and stored at −20°C until further use.

### 2.8. Cytokine Enzyme-Linked Immunosorbent Assay (ELISA)

For detection of the cytokines TNF-*α*, IL-10, IL-22, and TSLP in cell supernatants, human ELISA Ready-SET-Go! kits from eBioscience (San Diego, USA) were used according to the manufacturer's instructions. Here, IL-10 was detected with a sensitivity of 2 pg/mL, IL-22 and TSLP with a sensitivity of 8 pg/mL, and TNF-*α* with a sensitivity of 4 pg/mL.

### 2.9. Flow Cytometry

To measure the binding affinity of the* S. ratti* and* T. suis* Trx-lps to certain cell types, the purified proteins were labeled using the Alexa Fluor® 647 Protein Labeling Kit Microscale (A30009) from Invitrogen (Oregon, USA) according to the manufacturer's instructions. The binding affinity for both Trx-lps to monocytes, lymphocytes, and granulocytes from peripheral blood, as well as to the cell lines THP-1 cells (undifferentiated and differentiated) and Caco-2 cells, were tested. Approximately 2 × 10^5^ cells were used per reaction. The fluorescently labeled proteins were tested in four different concentrations (0.1 *μ*g and 0.2 *μ*g [data unpublished] and 0.4 *μ*g and 0.6 *μ*g). BSA labeled with Alexa Fluor® 647 was used as negative control. Each sample, which consisted of SrTrx-lp or TsTrx-lp and the cell type to be tested, was brought to a volume of 200 *μ*L with PBS and incubated for 30 min. All experimental setups were prepared in duplicate to test various temperatures. Incubation took place at RT (data not shown) and 37°C. After incubation, samples were washed twice, resuspended in 150 *μ*L PBS, and analyzed by flow cytometry on a FACScalibur cytometer (BD Biosciences), with 10,000 events collected from the gated populations. For further characterization of the binding specificity, cells were preincubated with 0.1 *μ*g and 0.2 *μ*g (data not shown) or 0.4 *μ*g and 0.6 *μ*g of unlabeled protein for 30 min prior to the addition of the corresponding labeled proteins. The data were analyzed with CellQuestPro.

### 2.10. Chemotaxis Assay

To evaluate the chemotactic activity of human monocytic THP-1 cells, Boyden chambers were used as described previously [68, 69]. DTT (100 mM) reduced Trx-lps from* S. ratti *and* T. suis* were tested at concentrations of 3 ng, 30 ng, 300 ng, and 1 *μ*g each in 100 *μ*L. The assay was performed with negative controls (random migration) such as chemotaxis buffer (PBS containing CaCl_2_, MgCl_2_, and BSA) and THP-1 media (RPMI containing HEPES and 10% FCS) and as positive control LPS at 100 ng, since LPS induces migration of monocytic cells [70]. THP-1 cells (2 × 10^5^) were allowed to migrate through polyvinyl-pyrrolidone-free polycarbonate filters (pore size: 3 *μ*m; Nuclepore, Tübingen, Germany) within 90 min at 37°C and 5% CO_2_. Afterwards, migrated cells were counted by using an inverted Zeiss microscope (Axiovert 25). Triplicates were performed in three independent experiments.

### 2.11. Wound Healing

To monitor epithelial cell migration of Caco-2 cells and the ability of Trx-lps to improve the wound healing process, we used the CytoSelect 24-Well Wound Healing Assay (Cell Biolabs, Inc.) according to the manufacturer's instructions. By means of the CytoSelect wound healing inserts a 0.9 mm wound field was generated. 500 *μ*L of a Caco-2 cell suspension (containing 0.5 × 10^6^ cells) was added to each well after the inserts had firm contact with the bottom of the wells. After overnight incubation, a monolayer was formed, the inserts were removed, the cells were washed, and the different stimuli were added. We used both Trx-lps, from* S. ratti* and* T. suis*, in concentrations of 3 ng, 30 ng, 300 ng, 1 *μ*g, 10 *μ*g, and 25 *μ*g per 500 *μ*L. As a positive control the human epidermal growth factor (EGF; 0.5 ng, 5 ng, 10 ng, 15 ng, and 25 ng) was included in order to get the proper concentration for maximal wound healing effects. As negative control cell media and LPS were added. An inverted digital microscope (EVOS™ FL Thermo Fisher Scientific) by Advanced Microscopy Group was used for observation (4x magnification). The cells were incubated for 4 days, whereby each 24 h a picture was taken and the percent closure was calculated.

### 2.12. Statistical Analysis

Statistical differences between groups were analyzed with the* t*-test for independent samples or the Mann–Whitney* U* test. *P* < 0.05 was taken as moderate evidence of significance and *P* < 0.01 as strong evidence of significance.

## 3. Results

### 3.1. Identification of Full-Length cDNAs Encoding the* S. ratti* and* T. suis* Trx-lps, Cloning, and Sequence Analyses

SrTrx-lp is represented by the cluster SR00399 [13] and was abundantly found in* S. ratti* E/S products of parasitic* S. ratti* females. The partial sequence was identified as the thioredoxin family protein and was used to obtain the full-length cDNA sequence by PCR. Further, the full-length cDNA sequence of the* T. suis* hypothetical protein M513 (Accession no. KFD58615.1) was cloned and identified as Trx-lp. The protein sequence of the recombinantly expressed* S. ratti* and* T. suis* Trx-lps have been verified by mass spectrometry.

Conserved domains of the Trx-lps from the intestinal helminths* S. ratti* and* T. suis* were ascertained by the protein families database (Pfam). Neither the Trx-lp from* S. ratti* nor the Trx-lp from* T. suis* contain a signal peptide. Both proteins have an N-terminal thioredoxin domain containing the active side motif CXXC (CGPC) and a C-terminal PITH (proteasome-interacting domain of thioredoxin-like) domain.

The alignment of the amino acid sequences from different organisms revealed a relatively low degree of identity between the different species. Between the Trx-lps from* S. ratti* and* T. suis* the degree of identity (39%) was not as high as between Trx-lps from* S. ratti* and* B. malayi* (56%). A high degree of identity was revealed between both* Trichuris* spp. Trx-lps (94%), similar to the sequences of* S. ratti* and* S. stercoralis* (99.9%) (data not shown). Comparing the other aligned helminth protein sequences, the similarities to the* S. ratti* and the* T. suis* Trx-lps varied between 35% and 56%. The comparison of the redox-regulating protein between* S. ratti* and* Homo sapiens* showed 43% identity.

**Figure 1:**
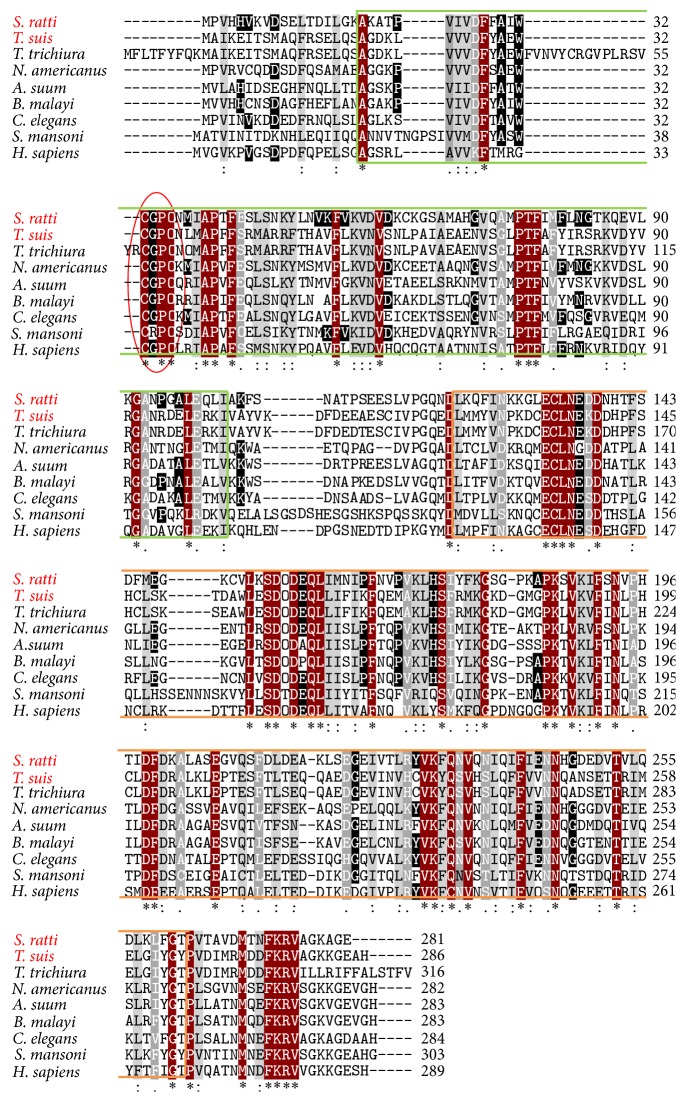
Multiple alignment of the Trx-lps from different organisms.* S. ratti* (CEF66761.1);* T. suis* (KHJ44020.1);* T. trichiura* (CDW52389.1);* Necator americanus* (XP_013304103.1);* Ascaris suum* (ERG80831.1);* Brugia malayi* (XP_001892562.1);* C. elegans* (NP_491127.1);* Schistosoma mansoni* (CD80891.1);* H. sapiens* (NP_004777.1). Green box represents the Trx-like domain; orange box represents the PITH domain; red circle shows the active site.

**Figure 2:**
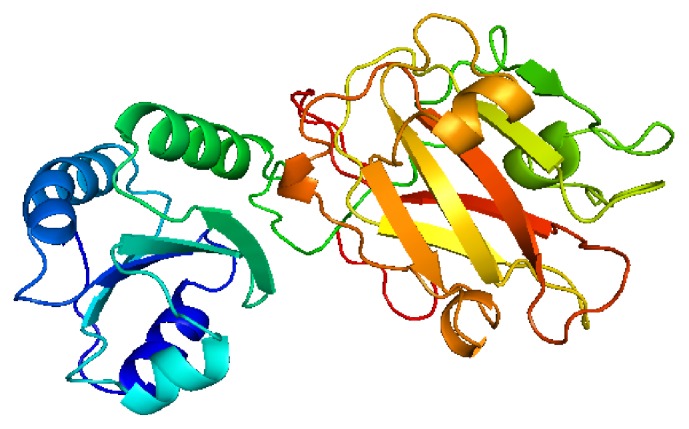
Predicted 3D-structure of parasite Trx-lp. The structure of SrTrx-lp is shown here. Both parasite Trx-lps have a Trx-like domain (left) as well as the PITH domain (right) (Phyre2: [55]).

The aligned helminth sequences share, except for the trematode* Schistosoma mansoni*, the catalytic site sequence (CGPC) with the human Trx-lp sequence of the active site. There are always two cysteines which are separated by two amino acids, mostly glycine and proline. Instead of a glycine, the* S. mansoni *catalytic site sequence has an arginine (R) (Figure 1). The two cysteines are responsible for the redox regulation in different cellular processes. The predicted structure of SrTrx-lp is exemplarily shown in Figure 2. Both parasite Trx-lps have a Trx-like domain (left) as well as the PITH domain (right) (Figure 2; Phyre2: [61]).

### 3.2. Recombinant Expression and Purification of* S. ratti* and* T. suis* Trx-lp

SrTrx-lp and TsTrx-lp were recombinantly expressed in endotoxin-free* E. coli* as His-tagged proteins and as strep-tagged proteins. The amount of purified His-tagged proteins, however, was higher than the amount of purified strep-tagged proteins. Thus, after preliminary tests with strep-tagged proteins, we further worked with His-tagged proteins. Both parasite proteins were verified by Western blot using anti-strep and anti-his antibody (Figure S1) and mass spectrometry.

### 3.3. Functional Activity Assays

#### 3.3.1. Reduction of Proteins

**Figure 3:**
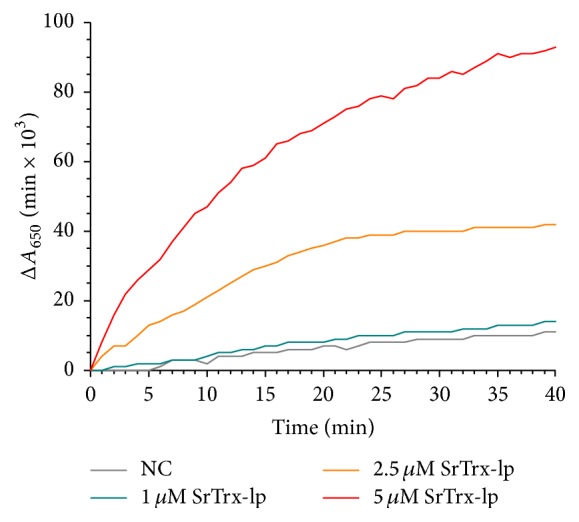
SrTrx-lp catalyzed reduction of insulin by DTT. Here, the rate of precipitation was plotted against time. While after 40 min the reduction of the SrTrx-lp was near the equilibrium, only a minor reduction of insulin was detected in the negative control (NC) without the SrTrx-lp and the lowest concentration used of SrTrx-lp (1 *μ*M).

*(1) Insulin Reduction*. For measurement of the functional activity of SrTrx-lp using insulin, the precipitation of free insulin *β*-chains was measured spectrophotometrically at a wavelength of 650 nm according to Holmgren (1979) as well as Luthman and Holmgren (1982) [61, 62]. A concentration of 1 *μ*M (30 *μ*g/mL), 2.5 *μ*M (75 *μ*g/mL), and 5 *μ*M (150 *μ*g/mL) of the SrTrx-lp was used and the measuring time was plotted against the rate of precipitation (Δ*A*_650_/min × 10^3^), which was about 0.064 Δ*A*_650_/min at the highest concentration. SrTrx-lp reduces insulin with a relative specific activity of 1556.67 and is regenerated by DTT whereby in the negative control and the lowest concentration of SrTrx-lp only a slight precipitation of insulin could be measured (Figure 3).

**Figure 4:**
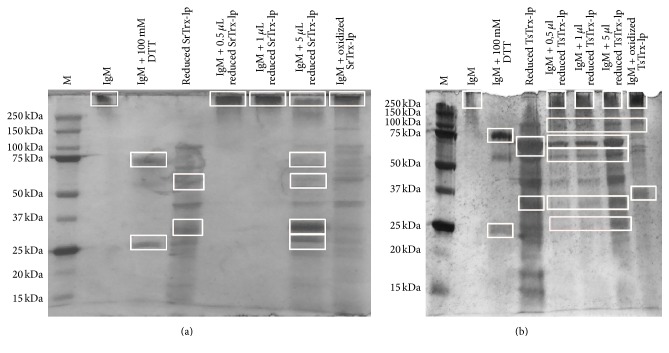
IgM reduction by the Trx-lps from* S. ratti* (a) and* T. suis* (b). Prior to incubation, the Trx-lps from both organisms were reduced by DTT. IgM was split in its chains (25 kDa, 70 kDa, and 950 kDa).

*(2) IgM Reduction*. Pentameric IgM consists of five M immunoglobulins joint by the J chain. Its molecular weight is about 950 kDa and it contains 26 interchain disulfide bridges that are potential substrates for Trx and thus for Trx-lp. Additionally to the insulin reduction activity assay, the dithiol-disulfide oxidoreductase activity of the Trx-lps was analyzed by an IgM reduction test according to Wollman et al. (1988) [63]. IgM is detectable at 250 kDa. As positive control IgM was reduced by 100 mM DTT at which bands at about 70 kDa (heavy chain IgM) and 25 kDa (light chain IgM) occur (Figure 4, 3rd lanes). Only exposing IgM to the highest amount of SrTrx-lp, five main bands were identified (Figure 4(a), lane 7). In addition to the bands at 70 kDa and 25 kDa, similar to the reduction of IgM with DTT, and the band at 250 kDa, now bands at about 30 kDa, representing monomeric* S. ratti* Trx-lp and 60 kDa representing dimeric* S. ratti *Trx-lp, were determined. A minor band was also seen at 45 kDa. Almost similar protein bands have been observed when* T. suis* Trx-lp was analyzed (Figure 4(b)); however,* T. suis* Trx-lp also at the low and intermediate concentration leads to the reduction of IgM. Further, bands at 140 kDa (heavy chain dimers of IgM) were predominant at all TsTrx-lp doses (Figure 4(b)).

### 3.4. Nematode Trx-lps Interact with Host Immune Cells

#### 3.4.1. Binding to Mucosal and Immune Cells

**Figure 5:**
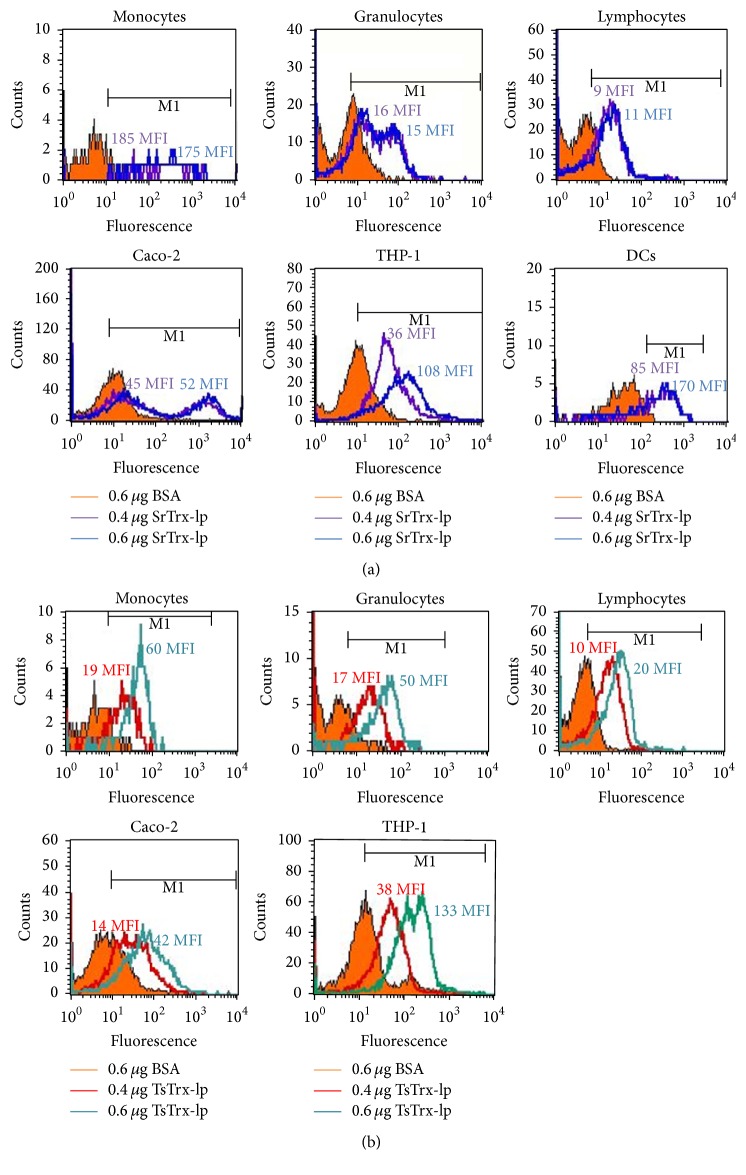
Binding of the SrTrx-lp (a) and TsTrx-lp (b) to different cell types. 2 × 10^5^ cells were incubated at 37°C for 30 min with Alexa Flour®-labeled SrTrx-lp or TsTrx-lp. Here, peripheral blood cells (monocytes, granulocytes, and lymphocytes) as well as cell culture cells (Caco-2 cells, THP-1 cells, and THP-1-derived DCs) were tested with 0.4 *μ*g (purple (a), red (b) line) and 0.6 *μ*g (blue (a), green (b) line) of labeled protein determining the median fluorescent intensity (MFI). The intensity of surface fluorescence (FI, *x*-axis) is plotted against cell counts. (The counts in the figures represent the median fluorescence index values.) Representative results of five independent experiments are shown.

The binding ability to other immune cells as well as mucosal Caco-2 cells was examined by FACS (Figure 5). Monocytes, lymphocytes, and neutrophils as well as Caco-2 cells, THP-1 cells, and THP-1-derived dendritic cells (DCs) were exposed to Alexa Flour-labeled Trx-lps. The experiments revealed considerable differential binding activities to various cells. Thus, SrTrx-lp (Figure 5(a)) as well as TsTrx-lp (Figure 5(b)) proteins strongly bound to monocytic cells shown in a dose-dependent manner for peripheral monocytes (SrTrx-lp: MFI 175–185; TsTrx-lp: MFI 19–60), THP-1 cell line (SrTrx-lp: MFI 36–108; TsTrx-lp: MFI 38–133), and generated DCs (SrTrx-lp: MFI 85–170). SrTrx-lp and at lower degree TsTrx-lp also bound to Caco-2 cells (SrTrx-lp: MFI 45–52; TsTrx-lp: MFI 14–42) and with limited affinity to neutrophilic granulocytes (SrTrx-lp: MFI 15-16; TsTrx-lp: MFI 17–50) and lymphocytes (SrTrx-lp: MFI 9–11; TsTrx-lp: MFI 10–20).

In order to verify the differentiation of THP-1 cells to DCs by IL-4 and GM-CSF, anti-CD86 antibodies were used. CD86 localized on the surface of differentiated DCs but not on THP-1 cells (data not shown).

#### 3.4.2. Nematode Trx-lps-Induced Cytokine Profile of Intestinal Epithelial-Dendritic Cell 3D-Cultures

**Figure 6:**
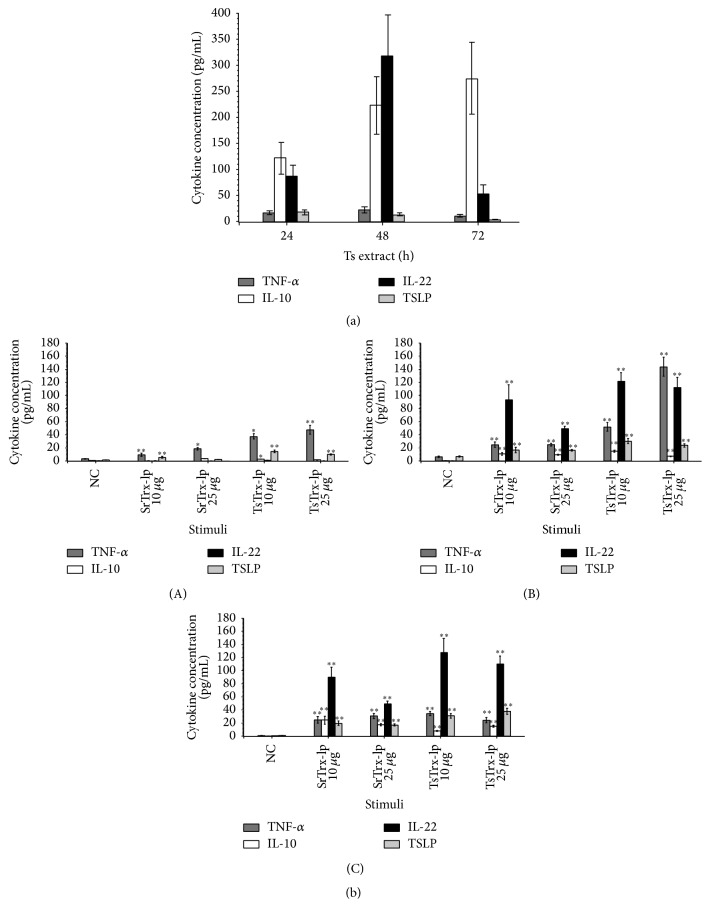
(a) Exposure of 3D-cocultures to* Trichuris suis* (Ts) extract. Culture supernatants were harvested after 24 h, 48 h, and 72 h. The release of inflammatory (TNF-*α*), anti-inflammatory (IL-10), and T_H_2-related cytokines (IL-22, TSLP) was analyzed in a 3D-cell culture model. Representative results of at least three independent experiments are shown as median. (b) Exposure of 3D-cocultures to SrTrx- and TsTrx-lp or medium (NC). Culture supernatants were harvested after 24 h (A), 48 h (B), and 72 h (C). The release of inflammatory (TNF-*α*), anti-inflammatory (IL-10), and T_H_2-related cytokines (IL-22, TSLP) was analyzed in a 3D-cell culture model. Representative results of at least three independent experiments are shown. Significant increase of all measured cytokines compared to NC (^*∗∗*^ *P* < 0.01). ^*∗*^ *P* < 0.05; ^*∗∗*^ *P* < 0.01.

The* S. ratti* and* T. suis* Trx-lps were examined for their ability to induce the release of cytokines in human 3D-cocultures of intestinal epithelial cells (IEC) and DCs. The release of the inflammatory (TNF-*α*), anti-inflammatory (IL-10), and T_H_2-related cytokines (IL-22, TSLP) was analyzed. In preliminary experiments, the optimized concentrations of LPS and LTA were determined as 0.5 *μ*g/mL and 0.1 *μ*g/mL (data not shown). 200 *µ*g/mL* T. suis* extract was used as a positive control and cell culture medium was used as a negative control (Figure 6(a)). The Trx-lps were tested at concentrations of 3 ng, 30 ng, 300 ng, 1 *μ*g, 10 *μ*g, and 25 *μ*g (each per mL). The reduced state (reduction via DTT) and the oxidized state (freshly purified protein, only partly reduced, see IgM reduction) of the Trx-lps made no difference in the cytokine response (data not shown). This observation indicated that the immune responses the proteins triggered are probably active site-independent. 10 *μ*g and 25 *μ*g of both helminthic Trx-lps are the most representative concentrations inducing the highest cytokine release.

Cocultured cells exposed to* T. suis* (Ts) extract showed in particular an enhanced production of IL-10 and IL-22 after 48 h and an even higher release of IL-10 after 72 h, while the proinflammatory cytokine TNF-*α* was downregulated (Figure 6(a)). SrTrx- as well as TsTrx-lp induced initially a slightly pronounced release of proinflammatory TNF-*α* after 24 h (*P* < 0.01), followed by an increased production of IL-22 and TSLP after 48 h of incubation (*P* < 0.01). In response to the exposure of the cocultures to Trx-lps in particular the T_H_2-associated cytokine IL-22 was produced after 48 h and 72 h (*P* < 0.01). At a concentration of 25 *μ*g of TsTrx-lp, the TNF-*α* release increased after 48 h and even dominated the IL-22 production. After 72 h, the IL-22 and TSLP production was dominating the overall TNF-*α* production. 10 *μ*g/mL of Trx-lps appears to be slightly more potent with respect to cytokine release than 25 *μ*g of protein with statistical significance only between the IL-22-inducing SrTrx-lp concentrations after 48 h (*P* < 0.01) (Figure 6(b)).

### 3.5. SrTrx/TsTrx-lp Displayed Chemotactic Activity for Monocytes

**Figure 7:**
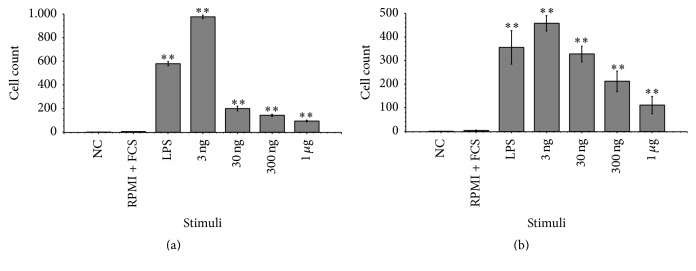
Chemotactic activity of the Trx-lp from* S. ratti* (a) and* T. suis* (b) for monocytic THP-1 cells. The chemotactic activity of both proteins for THP-1 cells was investigated by using Boyden chambers. Different concentrations of the Trx-lps were added to the lower compartment of the chemotactic chambers. Protein concentrations per 100 *μ*L of 3 ng, 30 ng, 300 ng, and 1 *μ*g showed SrTrx-lp and TsTrx-lp have the greatest chemotactic activity at 3 ng. Chemotaxis buffer (NC) and THP-1 media (RPMI + FCS) were included as negative control (random cell migration), while LPS was used as positive control. All used Trx-lp concentrations led to significant higher cell migration than the negative controls (^*∗∗*^ *P* < 0.01).

Human Trx is chemotactic for monocytes besides neutrophils and T lymphocytes [31]. Therefore, we investigated the chemotactic activity of the parasite Trx-lps for monocytic THP-1 cells by using Boyden chambers. Different Trx-lp concentrations (3 ng, 30 ng, 300 ng, and 1 *μ*g; each per 100 *μ*L) from both studied parasites were added to the lower compartment of the chambers. In the negative control, a few cells migrated through the membrane, while the cell migration using LPS as stimulant was significantly increased. Among the different applied Trx-lp concentrations the highest migration rate was detected at 3 ng. The overall cell migration was higher in case of* S. ratti* Trx-lp than after stimulation with the TsTrx-lp and half bell-shaped dose-response curve reported for chemokines is more pronounced in case of the TsTrx-lp (Figure 7).

### 3.6. Trx-lps Promoted Wound Healing

**Figure 8:**
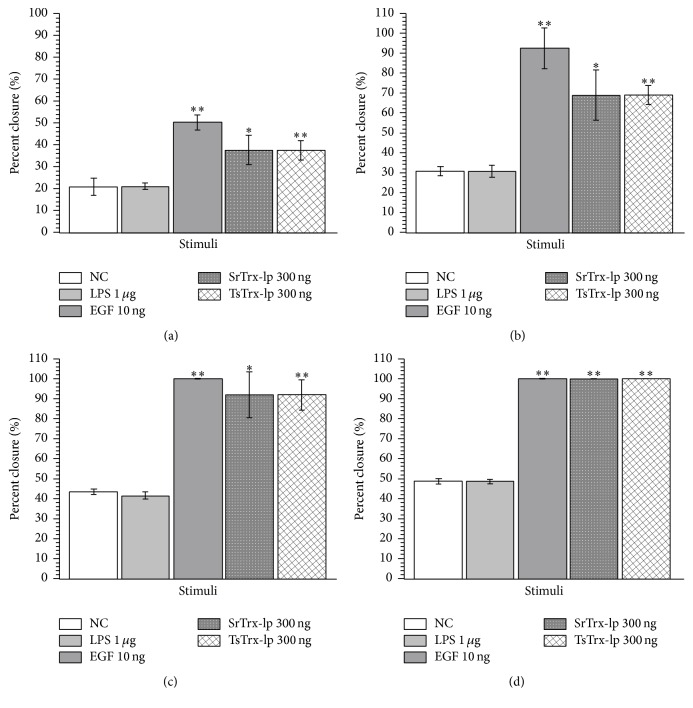
Percentage closure of the Caco-2 wound gap after 24 h (a), 48 h (b), 72 h (c), and 96 h (d). In general, the gap was narrowed by approx. 10–15% every day adding no stimulus. As a negative control, cells were observed without any stimulus (NC) and with LPS (1 *μ*g). As a positive control, epidermal growth factor (EGF) was used, whereat 10 ng fostered wound healing the best. SrTrx-lp and TsTrx-lp were tested at different concentrations (3 ng, 30 ng, 300 ng, 1 *μ*g, 10 *μ*g, and 25 *μ*g–each per 500 *μ*L), here at 300 ng represented as the best wound healing promoting concentration. The wound healing process was highly significantly promoted by EGF and TsTrx-lp (^*∗∗*^ *P* < 0.01) as well as significantly promoted by SrTrx-lp (^*∗*^ *P* < 0.05).

**Figure 9:**
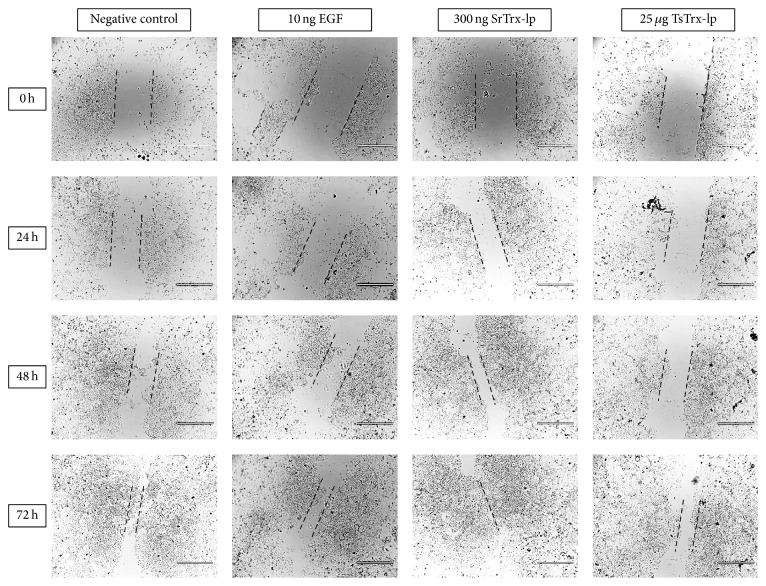
Wound healing assay with Caco-2 cells and the* S. ratti* as well as the* T. suis *Trx-lp. The CytoSelect™ 24-Well Wound healing assay was performed. Here, two examples are described as representatives for different tested concentrations. Protein concentrations of 300 ng SrTrx-lp are given as example for best concentration for wound healing promotion and 25 *μ*g of TsTrx-lp (each per 500 *μ*L) indicated the less wound healing-promoting concentration. The wound healing process of a 0.9 mm wound field generated was observed over 96 h, whereby each 24 h a picture was taken. The size of the scale bar is 1000 *μ*m and the dashed black lines indicate wound-like area [56].

As an important functional activity it was investigated whether the Trx-lps from both nematode parasites express wound healing activity. Therefore, the effect of different concentrations of Trx-lps on epithelial cell (Caco-2) wound closure (Figure 8, data, and Figure 9, microscopic photography) was analyzed. Compared to the untreated cells, where the wound-like area narrowed 10–15% every day, the stimulated cells showed almost twice as much growth. 300 ng/500 *μ*L of both parasite Trx-lps are the most potent concentration for promoting the wound healing process as well as 10 ng of EGF, which was included as positive control, while 3 ng and 30 ng and concentrations upon 1 *μ*g (each per 500 *μ*L) have a more moderate effect on wound healing. The wound healing process was highly significantly promoted by EGF and TsTrx-lp (^*∗∗*^ *P* < 0.01) as well as significantly promoted by SrTrx-lp (^*∗*^ *P* < 0.05).

## 4. Discussion

Trx is a physiologically important multifunctional protein and prokaryotic Trx has been described as so-called moonlighting protein [34, 35]. The multiple biological functions comprise features as growth factor and antioxidant, as inhibitor of apoptosis and transcriptional factor, and as chemokine [22, 23, 25–28]. Very little is known about Trx-lps, in particular about those from helminths and their potential role in parasite-host interaction.

There is only one publication about an endoplasmic reticulum located Trx transmembrane related protein from the trematode* Clonorchis sinensis*, containing a Trx domain with the active site motif Cys-Pro-Ala-Cys (CPAC). This redox molecule is suggested to serve as protection against host- and parasite-generated ROS [44].

Contrariwise, the* S. ratti* Trx-lp has the catalytic domain sequence of the uniformly small (12 kDa) ubiquitous Trx proteins (WCGPC) but has a size of approximately 30 kDa. Comparably, the* T. suis* Trx-lp has a size of approximately 33 kDa and the same catalytic domain sequence as the classic Trx.

In the present study, Trx-lp from two parasitic nematodes,* S. ratti* and* T. suis*, were cloned, expressed, and characterized for the first time. In case of both helminths the protein was present in the E/S products of the parasites [13, Brattig et al., unpublished]. The molecular mass (30–33 kDa) as well as the proteins structure suggested similar functions to those of the human Trx-related protein (TRP32), also known as TXNL-1, which protects the cell against glucose deprivation-induced cytotoxicity and is involved in antiapoptotic signaling [47, 48, 71]. Like SrTrx- and TsTrx-lp, TRP32 consists of an N-terminal Trx and a C-terminal PITH domain as well [44]. Trx-lps are known to have several binding partners and substrates they associate with by means of their Trx domain, which exerts redox-active functions. The C-terminal PITH domain is able to interact with the 26S proteasome by the substrate-recruiting factor of the 26S proteasome eEF1A1 [72, 73].

Similar to Trx, Trx-lps of eukaryotic cells are also multifunctional and involved in different cellular processes including cofactor functions or the regulation of specific signaling proteins [46] which may indicate possible moonlighting properties that have to be demonstrated in the future [34–36]. Comparisons of Trx-like homologues by multiple sequence alignments revealed a high sequence similarity between Trx-lps from* T. suis* and from* T. trichiura *(94% identity).* Strongyloides* species are all very closely related [11, 74]. Apart from this, the protein alignment showed a relatively low degree of similarity (35%–56%) between different nematodes, either parasitic or nonparasitic. Except for* S. mansoni* all other species had the strongly conserved N-terminal Trx catalytic site sequence (CGPC). At the C-terminus all Trx-lps possess the PITH domain. Like Trx, the analyzed parasite proteins have no signal peptide and are released from cells by nonclassical protein export [29, 75].

Trx-lp has also various roles in several human cellular and extracellular processes, since reactive oxygen species (ROS) occur in the normally functioning metabolism [76]. The dithiol-disulphide oxidoreductase activity of both recombinant* S. ratti* and* T. suis* Trx-lps was either analyzed by insulin reduction according to Holmgren (1979) or IgM reduction according to Wollman et al. (1988) [61, 63]. Reduced Trx reacted very quickly with insulin and the reduced insulin was precipitated. The relative specific activity of Trx from* E. coli *amounts to a value of 4930 units [61]. Findings that measured relative specific activity of the SrTrx-lp has an activity of about 1557 units show that it has a comparable activity to classical Trx. The oxidoreductase activity was further analyzed by the reduction of murine IgM. Wollman et al. (1988) have already shown that recombinant human Trx is able to reduce the disulfide bridges of murine IgM [63]. Therefore, we suggested Trx domain containing Trx-lps may also have the ability to reduce IgM. We could show that indeed both Trx-lps reduced the S-S bonds of IgM. Since all TsTrx-lp used doses resulted in the formation of the same bands in SDS-PAGE, this Trx-lp appears to be more active than the* S. ratti* Trx-lp. Even at the lowest concentration minor protein bands were visible at 25 kDa and 70 kDa. The more intensive they were the higher the added concentration of TsTrx-lp was. A reduction of IgM by not fully removed DTT can be excluded since then the strength of the formed bands would be the same in each approach. Although even at the lowest concentration bands have been formed, they were more intensive at the highest concentration. Furthermore, in the IgM reduction assay of SrTrx-lp no bands were existent at the lowest and the intermediate concentration of the added protein.

Through those activity assays it could be demonstrated that the recombinantly expressed Trx-lps have redox functions and are able to act as classical Trx. In further analysis, we could demonstrate multifunctional activities of the helminth proteins. For Trx it has been reported to be released by monocytes [77] and also to be chemotactic for monocytes, neutrophils, and T lymphocytes [31]. Accordingly, we have observed that* S. ratti* and* T. suis* Trx-lps exhibit chemotactic activity for monocytes and have the ability to interact with them. An attraction of monocytic cells to a nematode-dwelling site could subsequently lead to an activation of the cells leading to a consecutive generation of wound healing fostering cytokines like IL-22 and immunoregulatory interleukins [78–80]. Both parasite Trx-lps bound to monocytic cells, to the THP-1 cells, and to peripheral monocytes although in some FACS analysis there were only limited counting events. Accordingly, SrTrx-lp was shown to bind to DCs. Of interest, the parasite redox-regulating proteins also bound to Caco-2 cells and more weakly to lymphocytes and granulocytes. Thus, Trx-lps seem to interact with intestinal epithelial cells, the first-line host cells that get exposed to E/S products released by the colonizing parasitic females, and also with second-line cells, the monocyte-derived DCs.

Of interest, Trx has been reported to possess immunological activities. Thus, it has been attributed to an anti-inflammatory role besides suppression of apoptosis and fostering cell growth [32, 81–83]. Trx can interact with immune cells and facilitates the production of TNF-*α* [31, 84] by monocytic lineage, but it is also able to counteract the production of inflammatory cytokines such as TNF-*α* [85, 86]. In the present study, 3D-coculturing of the intestinal epithelial Caco-2 cells and THP-1-derived DCs was performed. Hereby, parasite Trx-lps induced the release of proinflammatory TNF-*α* in the first day of the culture and at high concentration after 48 h followed by a prevailing generation of the T_H_2-related cytokine IL-22 besides lower levels of TSLP and IL-10. IL-22 may be predominantly released by activated DCs in the cell cultures after 2-3 days [78, 80, 87].

IL-22, particularly produced by immune cells present beneath the epithelium, as the innate lymphoid cells [78, 80, 88], acts through signal transducer and activator of transcription (STAT-3) and is important in maintaining the homeostasis of the gut and therefore serves the protection from intestinal inflammation. An important source of IL-22 in acute colitis is TLR-stimulated CD11c^+^ DCs which are located in the surficial mucosal epithelium of the gut and are getting activated by invading pathogens like bacteria or parasites. These cells initiate, via IL-22 and thus STAT-3, processes that are important for a proper stress response, mucosal wound healing, and apoptosis pathway [78, 79, 89]. IL-22 may profoundly increase the proliferation and turnover of IECs and the production of mucus and antimicrobial peptides [90]. Accordingly, the release of proteins from intestinal nematodes like Trx-lps may contribute to preserve or restore the integrity of the intestinal barrier.

Thus, there are three possible pathways for helminthic Trx-lps to act: firstly, secreted Trx/Trx-lp protects the parasite against high ROS production initiated by the host's first-line immune response via cells of the monocyte-macrophage linage. Trx may be important for redox control at wound margins, since much ROS emergence was proven there [91, 92]. ROS as second messenger ameliorates wound healing processes [93]. Therefore, among others, it serves the migration of cells and closure of wounds. Then, antioxidant molecules are probably important to maintain the balance in order to prevent stress-induced cell death. Secondly, secreted Trx-lp stimulates mucosal DCs to generate high levels of IL-22 which promotes epithelial cell proliferation and the preservation or restitution of the integrity of the intestinal barrier. In the present study we had shown that 300 ng of parasite Trx-lps promoted the wound healing process of epithelial Caco-2 cells. A third possible function of Trx-lp secreted by the parasite may be to mimic antioxidant molecules of the host and may lead to interference reactions in the host's antioxidant metabolism concerning the substrates and binding molecules. Thus, recent reports indicated that distinct molecules secreted by helminth parasites can foster wound healing [94] and modulate the host's immune response [95].

## 5. Conclusion

In summary, we identified and characterized the secreted Trx-lps from* S. ratti* and* T. suis*. Both multifunctional proteins expressed antioxidative activity and the capability to interact with the host's mucosal cells, indicated by chemotactic activity for monocytic cells, binding to host's epithelial cells as well as to immune cells, by the release of cytokines. In particular, the promoting wound healing effect indicates the involvement of Trx-lp in many pathways that are initiated in the local parasite-host interaction

## Supplementary Material

In the Supplementary Material pictures of SDS-PAGE (protein purification) and Western Blot analysis of the recombinantly expressed SrTrx-lp and TsTrx-lp are provided.
Coomassie-stained SDS-PAGE of purification steps of recombinant SrTrx-lp (left) and TsTrx-lp (right) as well as Western Blot analysis (middle) of both eluted proteins. Elutions (E1-E5) were analyzed by Western Blot using anti-his antibody. There were distinct lines at approximately 33 kDa (SrTrx-lp) and 36 kDa (TsTrx-lp). P: pellet; FT: flow through; W1: wash step 1; W2: wash step 2.
