# Supplementary material for: Multifunctional Thioredoxin-Like Protein from the Gastrointestinal Parasitic Nematodes Strongyloides ratti and Trichuris suis Affects Mucosal Homeostasis
Source: J Parasitol Res. 2016 Oct 31;2016:8421597. doi: 10.1155/2016/8421597 (PMC5107843; doi:10.1155/2016/8421597)
Supplement: Supplementary file 1 — In the Supplementary Material pictures of SDS-PAGE (protein purification) and Western Blot analysis of the recombinantly expressed SrTrx-lp and TsTrx-lp are provided. Coomassie-stained SDS-PAGE of purification steps of recombinant SrTrx-lp (left) and TsTrx-lp (right) as well as Western Blot analysis (middle) of both eluted proteins. Elutions (E1-E5) were analyzed by Western Blot using anti-his antibody. There were distinct lines at approximately 33 kDa (SrTrx-lp) and 36 kDa (TsTrx-lp). P: pellet; FT: flow through; W1: wash step 1; W2: wash step 2. [file 8421597.f1.pdf]

Supplementary Material

In the Supplementary Material pictures of SDS-PAGE (protein purification) and Western Blot analysis of the recombinantly expressed SrTrx-lp and TsTrx-lp are provided (Supplementary Figure 1).

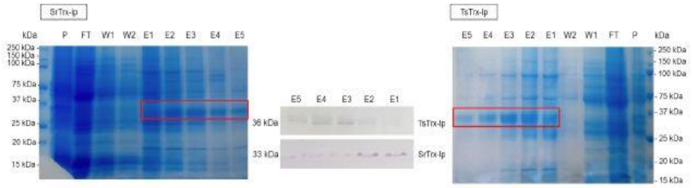

SUPPLEMENTARY FIGURE 1: Coomassie-stained SDS-PAGE of purification steps of recombinant SrTrx-lp (left) and TsTrx-lp (right) as well as Western Blot analysis (middle) of both eluted proteins. Elutions (E1-E5) were distinct lines at approximately 33 kDa (SrTrx-lp) and 36 kDa (TsTrx-lp). P: pellet; FT: flow through; W1: wash step 1; W2: wash step 2.
